# Evolving Treatment Options for Spinal Pain: Balancing Innovation and Affordability in Regenerative Medicine

**DOI:** 10.1007/s11916-026-01552-x

**Published:** 2026-09-03

**Authors:** Annu H. Navani, Sudhir A. Diwan, Douglas P. Beall, Edward S. Rubin, Ahmed I. Anwar, Alan D. Kaye

**Affiliations:** 1 Department of Research and Development, Le Reve Regenerative Wellness, San Jose, CA USA; 2 Boomerang Healthcare, Walnut Creek, CA USA; 3 Albert Einstein College of Medicine, New York, NY USA; 4 Advanced Spine on Park Ave, New York, NY USA; 5 Comprehensive Specialty Care, Edmond, OK USA; 6 Edward Rubin MD Pain Management, 1103 Stewart Ave, 3rd Floor, Garden City, NY 11530 USA; 7 Department of Anesthesiology, Louisiana State University Health Shreveport, Shreveport, LA 71103 USA

**Keywords:** Back pain, Cost-effectiveness of regenerative spinal procedures, Degenerative disc disease, Discogenic disease, Low back pain, Mesenchymal stem cells, Platelet-rich plasma, Spinal pain, Pain medicine

## Abstract

**Purpose of Review:**

Regenerative approaches for treating spinal pain hold great clinical promise but have the potential to disrupt healthcare upending the cost paradigm of pharmacologic care or interventional procedures. Regenerative approaches have higher upfront costs but may offer long-lasting, even curative benefits. In today’s healthcare system, it is important to understand the cost-effectiveness of regenerative approaches using mesenchymal stem cells (MSC), platelet-rich plasma (PRP), and other biologics. This narrative review explored the costs of these procedures for treating spinal pain, but since this specific indication is not currently approved for clinical use in the United States, cost data were sparse.

**Recent Findings:**

This study was based on a narrative review and data obtained from the literature and available to the authors from a scientific session. While regenerative medicine is approved for certain indications, it has not yet been approved for use to treat painful spinal conditions, although there is evidence that it is a safe and effective procedure in this setting. In fact, PRP approaches to other musculoskeletal painful conditions are approved and increasing, according to Medicare. When compared to fusion surgery, MSC treatment of discogenic disease cost approximately $11,000 in the United States compared to two years of epidural spinal injections costing approximately $900 each; the regenerative approaches offered better pain reduction (66% vs. 35% to 51%). Spinal fusion surgery runs $79,595 to $105,657 according to current procedural terminology. Once regenerative approaches for the spine are approved, there may be more data to provide greater insight into cost-effectiveness analyses.

**Summary:**

There is a paucity of cost data and since these procedures are off label, these figures are subject to change and may vary regionally. Regenerative medicine offers the potential to greatly advance the treatment of back pain, but in today’s cost-conscious healthcare system, cost considerations must be weighed along with efficacy and safety data.

## Introduction

Back pain treatments have changed little in 40 years despite an urgent, growing, and unmet medical need for better control of spinal pain [1]. Regenerative approaches for treating spinal pain lack approval for use in clinical practice in the United States, but represent an emerging, important, and potentially disruptive paradigm for clinical practice, because regenerative medicine relies on higher initial costs but with the promise of long-term benefits. Regenerative medicine practice guidelines state that for certain chronic pain conditions, the use of injectable biologics may offer better analgesia, improved function, and superior quality of life when compared to conventional medical treatments or placebo [2]. The challenge today is how to best define, guide, and integrate regenerative medicine into modern cost-constrained clinical practice in an aging population.

The use of mesenchymal stem cells (MSC), platelet-rich plasma (PRP), acellular and other biologic substances in regenerative procedures may be helpful not only to control spinal pain, but also by offering disease-modifying attributes [3]. While regenerative procedures are approved by the U.S. Food and Drug Administration (FDA) for certain types of large cell B-cell lymphomas in 2017 and sickle cell disease in 2023, their promising role in spinal pain management remains under investigation [4–6]. This relegates regenerative procedures to control spinal pain to off-label status, meaning there are only limited clinical trials or reports in the literature about these procedures. Cost data from academic literature is sparse. The wide variety of biologics, numerous spinal indications and non-uniformity in use of orthobiologics for spinal pain add to the heterogeneity of comparing specific procedures.

Global lifetime prevalence of low back pain is very high, approaching 40%, and as certain regions of the world age, this population will increase, thereby bolstering its status as the most important cause of disability around the world [7, 8]. Corticosteroid injections and other interventional pain procedures are common for selected patients, but may only confer short-term benefits [9]. Pharmacologic therapy based on the foundation of opioids has come under increased scrutiny, in part because of the known drawbacks of opioids including tolerance, side effects, endocrine system disruptions, and opioid-induced hyperalgesia [10–13]. The lack of an effective treatment option for long-term results presents an opportunity for orthobiologics. Orthobiologics carry the promise of repair and regeneration while addressing the pathology itself.

In today’s increasingly cost-constrained national and global healthcare systems, cost-effectiveness data can play an outsized role in treatment choices. Unfortunately, head-to-head comparisons between conventional versus regenerative approaches to spinal pain control are uncommon, but a broad view can be taken to explore whether regenerative approaches are safe, effective, provide durable results, and if costs can be balanced against treatment outcomes. It has been shown that regenerative medicine can improve painful symptoms of spinal conditions [2]. The straightforward and low-risk regenerative approaches can appeal to patients otherwise faced with spinal surgery or long-term drug treatment, but cost must be considered. Cost effectiveness in medicine refers to the cost as measured against a specific outcome, such as cost of a life saved. Although these terms are sometimes conflated, cost utility refers to the cost as measured against both a quantitative outcome as well as quality of life, such as the metric quality-adjusted life year. Cost utility assessments are often more helpful in terms of clinical decision-making processes [14]. In our narrative review we reported results as the studies reported them, although in general cost utility assessments are more informative for healthcare systems than cost-effectiveness data alone.

Cost-effectiveness can be challenging to assess in medicine, since it can be difficult to quantify a price for pain relief, restored function, patient satisfaction, and enhanced quality of life. Furthermore, regenerative approaches to spinal pain are new and evolving; there is no “gold standard” of treatment and no official guidelines regarding which biologics and techniques are most appropriate for specific indications. Thus, evaluating safety, effectiveness, and cost-effectiveness for regenerative medical techniques to ameliorate spinal pain must be considered in a broad context until more specific expert consensus guidelines are issued.

The aim of this narrative review, therefore, is to help interpret cost effectiveness of regenerative approaches for back pain compared to conventional approaches, even with limited data currently available.

## Methods

On January 13, 2025, keywords were searched in the PubMed database, the Cochrane Library, and Google Scholar. For Google Scholar, the first 20 results were taken. “Regenerative medicine, spinal pain, cost effective” (11, 1, 20 articles, respectively), “regenerative medicine, spinal pain, cost effectiveness (1, 4, 20), “regenerative medicine, spine, cost effective” (1, 20, 23), “regenerative medicine, spine, cost effectiveness” (1, 8, 20), “stem cell, spinal pain, cost effectiveness” (0, 3, 20), “stem cell, spinal pain, cost effective,” (3, 9, 20), “orthobiologic, spinal pain, cost effectiveness” (0, 2, 20), and “orthobiologic, spinal pain, cost effective” (1, 2, 20). In total, 230 articles were retrieved in these searches. There was considerable duplication and when duplicates were removed, there was a total of 113 items. The authors also identified a presentation made at a specialty society which was also included (n = 1), making 114 items.

The inclusion criteria for the search were that the material be in English and relate to the cost or cost-effectiveness of orthobiologic procedures with respect to spinal pain. Excluded were clinical trials that did not address costs, patents, editorials, and case studies. Articles about materials cost, production costs, or other cost factors not related directly to treating patients were excluded. No delimiters were used in the search with respect to article dates, but articles published more than five years earlier (cutoff date: prior to 2020) were not used for cost data but were still examined in for other related information. The bibliographies of articles were searched and relevant articles were reviewed, as was gray literature. Book chapters, conference proceedings, and abstracts were included; individual case studies, editorials, and dissertations were excluded. Conference proceedings, consensus guidelines, and cost information from commercial companies available online were also reviewed.

**Fig. 1:**
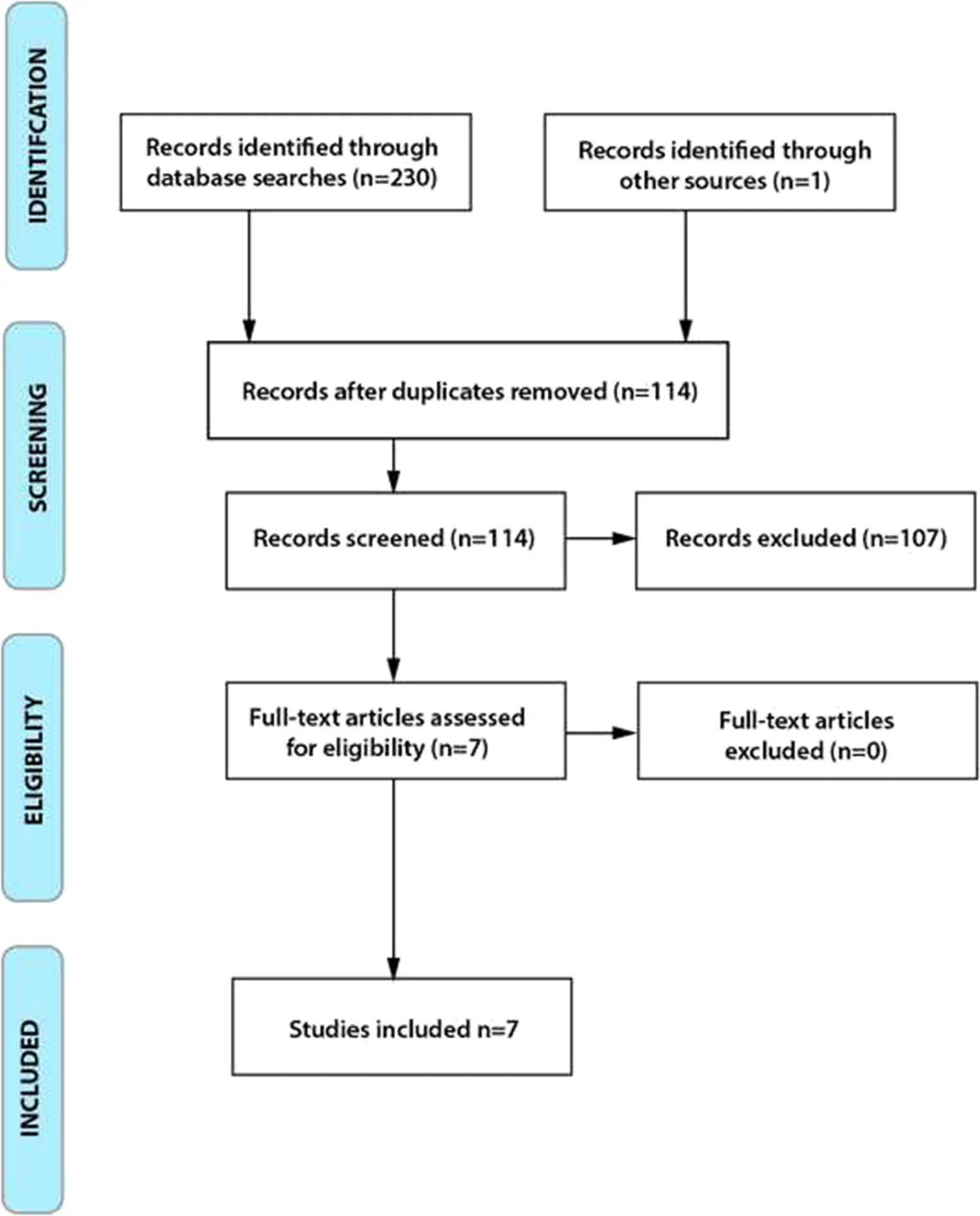
Description of literature search. Note that the literature search yielded 113 items and a presentation from a specialty society was added (*n* = 1) making 114 results total, of which 113 came from the literature search

The abstracts of these 113 items were reviewed 107 were excluded because they did not have cost data, did not include spinal procedures, or were about materials costs rather than clinical costs. The presentation from the specialty society (*n* = 1) did not have an abstract. This left 6 articles. This is a narrative and not a systematic review, nevertheless a chart modeled after a PRISMA systematic review chart is included to better illustrate the search efforts (Fig. 1).

## Results

There remains no first-line “gold standard” approach to spinal pain, leaving clinicians to make treatment choices based on available guidelines, the existing body of literature and evidence, as well as their own clinical experience and patient preferences. Regenerative medicine for spinal care represents a paradigm shift in the treatment of back pain, because it addresses the underlying cause of low back pain, using potentially disease-modifying effects rather than solely ameliorating symptoms [15, 16]. Regenerative approaches are appropriate to utilize early in the course of the disease [17]. A variety of biologics are in widespread use (Table 1).

**Table 1:** Orthobiologics and their putative mechanisms of action [2, 16, 18, 19]

Substance	Description	Mechanism
Adipose-derived mesenchymal stem cells (ADSCs); this includes stromal vascular fractions (SVF)	ADSCs are a type of MSC obtained via lipo-aspiration from adipose tissue.	Not fully elucidated but likely involves secretion of cytokines and growth factors; ADSCs release free radical scavengers and has an antioxidant effect
Bone marrow aspirate concentrate (BMAC), also known as bone marrow concentrate (BMC) that carry MSC	BMAC is a substance derived from bone marrow aspiration, which includes MSC	Promotes growth factors and has an anabolic and anti-inflammatory effect
Allogenic MSCs that can be obtained from a variety of sources, including, umbilical cords, bone marrow, etc.	MSCs can proliferate and differentiate into cells which can help regenerate tissue	MSCs express growth factors which stimulate local tissue repair while suppressing inflammatory T-cells and monocyte maturation. They decrease inflammation and reduce pain
Platelet-rich plasma (PRP)	Autologous platelets at high concentrations with other cell material removed	PRP initiates the body’s natural repair mechanisms, reduces inflammation, delivers growth factors, activates MSCs, and promotes a pro-healing environment
Induced pluripotent stem cells	Mature cells thar are genetically transformed into pluripotency with the ability to differentiate into multiple cell lineages	Stem cells differentiate and replicate to regenerate diseased or damaged tissues

*ACSC*, adipose-derived signaling cells; *BMAC*, bone marrow aspirate concentrate; *MSC*, mesenchymal stem cells; *PRP*, platelet-rich plasma; *SVF*, stromal vascular fraction

The American Society of Interventional Pain Physicians (ASIPP) has described biologics as a more economical approach that expedites healing and states that biologics are becoming a reasonable alternative for patients who fail standard-of-care treatments [15]. The American Society of Pain and Neuroscience (ASPN) has published evidence-based clinical guidelines for interventional treatments of low back pain, describing regenerative therapies for degenerative disc disorders as safe, overall, with complications comparable and not worse than those of other types of intradiscal procedures [19]. The reported complications following regenerative procedures for degenerative disc disorder were rare, but included back pain, muscle spasms, and discitis [19]. Other studies have found that the use of MSC for treatment of pain following spinal cord injury reported no serious treatment-related adverse events [20, 21].

Regenerative approaches for the treatment of spinal pain, in particular intradiscal PRP and MSC therapy, have been reported in the literature as safe, effective, and associated with few and mild side effects [18, 21–27]. The durability of the positive results seen with intradiscal treatments have been reported five to nine years after the procedure [28].

Since regenerative medicine has not yet been approved for the treatment of spinal pain, cost-effectiveness data are infrequent and incomplete [29]. Comparative studies do not exist and reliable national data on costs of regenerative procedures are not available. Without established payer data, it is difficult to know the actual cost of various regenerative procedures.

The United States spends approximately $90 billion annually on low back pain, making it one of the most expensive conditions to treat in American medicine. This $90 billion reflects costs for diagnosis and treatment only and does not address an additional $10 to $20 billion in lost productivity costs [30]. These are the best data available, and they are at least 10 years old, so these costs must be presumed to be lower than current spending levels. Based on these data, the treatment for discogenic low back pain, which is the most common form of low back pain accounting for approximately 39% of all cases of chronic low back pain, would amount to a little over $35 billion even with conservative estimates [31, 32]. Thus, if regenerative approaches for discogenic back pain can provide clinically meaningful pain control, excellent safety, and good tolerability at a reduced cost compared to conventional approaches such as open or minimally invasive surgery, this could represent a marked cost savings with equivalent or possibly superior outcomes.

Spinal fusion and other open-surgical procedures are associated with high upfront costs, adverse events, potential complications, and the risk of adjacent segment disease [33]. Interventional pain procedures, such as corticosteroid injections, are less expensive but may offer only short-term benefits and must be repeated. Pharmacologic therapy is relatively inexpensive upfront, but medications must be taken on an ongoing basis for the continuation of symptom relief. Thus, it is difficult to directly compare such conventional procedural costs to regenerative procedures, which have high upfront costs but may provide long-term value over time [34]. Indeed, the model for regenerative therapy is inherently disruptive to the current clinical paradigm, because it offers potentially long-term benefits with a single treatment and may reduce or eliminate the need for analgesics. Surgical approaches often require long-term pharmacologic supplementation to maintain analgesic benefit. Regenerative medicine, on the other hand, may suffice with one treatment; it may even be curative.

While these promising potential benefits are important, they challenge current models of cost versus benefit analysis. Since regenerative approaches are novel emerging therapies for which the regulatory, legal, and reimbursement issues have not yet been clarified, differences among national and regional healthcare systems may mean that regenerative medicine for spinal pain may reach some populations earlier than others. At this time, regenerative medicine overall is used mainly in North America (53%), Europe including Israel (26%), and in Asia (16%), with very low reported use elsewhere [34]. This means that cost-effectiveness data must be viewed through a national or regional lens. Product evolution is rapid in this space, further adding to the variations in terms of the optimal approach for a given patient.

The lack of Food and Drug Administration (FDA) approval means that regenerative procedures in the United States are typically paid out-of-pocket and their limited availability may contribute to higher prices. The lack of reimbursement data provides a paucity of reliable real-world information on costs. In a survey of 100 physicians who reported performing regenerative procedures, MSCs from autologous bone marrow were more expensive than PRP procedures, as might be expected [29]. Older data from 2010 to 2011 and published in a 2016 article reported on billing records for 2,571 patients who had PRP injections for a variety of musculoskeletal disorders. In this study, the average per-patient charge in the United States per PRP treatment was $1,755 per injection [35].

Since regenerative procedures must be paid out-of-pocket in the United States, some healthcare centers have posted pricing on their websites: a clinic in Minneapolis advertises that stem cell treatments cost between $3,500 to $6,000 [36], a clinic in Fort Worth states that stem cells for orthopedic use cost $4,500 for a single injection and $5,000 for two injections [37], while a Boston provider states stem cell treatments for orthopedic use cost from $1,300 to $8,500 [38]. Obviously, costs vary by the clinical diagnosis, type of orthopedic procedure, how the cells are sourced, and the location of the laboratory. Many of these providers offer treatment in an ambulatory center.

A retrospective epidemiologic study based on Medicare data reported that PRP approaches for all types of musculoskeletal pain were increasing markedly. A commercial administrative claims database study found similar increases (Table 2).

**Table 2:** PRP treatment for musculoskeletal pain from a Medicare and a commercial administrative claims database

Source	Years	Aggregate charges for PRP injections	Units	Cost per injection
Medicare database	2010–2014	$2,000,000	3,654 injections	$547.35
Commercial database	2010–2020	$683,974 nonsurgical costs	17,759 treatments	$38.51

The total number of PRP treatments in the Medicare database increased 400% from 2010 to 2014 [39, 40]

Although not specific to spinal pain treatments, a study from Iran compared PRP to plasma-rich growth factor (PRGF), ozone, and hyaluronic acid (HA) in the treatment of pain related to knee osteoarthritis and found PRP to be a cost-effective therapy [41–48]. In this study, the cost for one intra-articular injection was $103 for ozone, $319 for PRP, $328 for PRGF, and $582 for HA and patients reported these procedures were of great value to them [41–48]. There was no comparable study for regenerative approaches to spinal pain, and this study came from the Iranian healthcare system, where pricing differs markedly from North America and Europe.

Regenerative procedures offer several advantages that would suggest cost reduction: there are minimal and often no side effects, recovery time is brief, healing is rapid, and the amount of inflammation is reduced. Some regenerative procedures can be done on an outpatient basis and, if required, a hospital stay would likely be brief. Regenerative treatments are less invasive than surgery, there is no need for opioids for postoperative analgesia, and patients overall seem favorably inclined to the convenience of these techniques compared to surgery. A recent conference detailed regenerative treatments in terms of cost, pain reduction, visual analog scale (VAS) pain measurements, and changes in the Oswestry Disability Index (ODI). See Table 3.

**Table 3:** Discogenic disease treatment using stem cells versus fusion surgery [41–48]

Treatment	Cost	Mean Pain Reduction %	VAS Improvement	ODI Improvement
Stem cell augmentation of IVD	$11,093.58	66%	54%−61% At 3 years	51%−55% at 3 years
Fusion Surgery CPT 22612) or disc arthroplasty Single-level fusion, single segment	$79,595 to $105,657	18% to 33%	27–33% at ≥ 4 years	22–33% at ≥ 4 years
Epidural spinal injections (two years, 8 injections, $889 each)	$7,112 to $9,245	35% to 51% at one year 49% at two years	--	--

*CPT*, current procedural terminology; *IVD*, intervertebral disc; *ODI*, Oswestry Disability Index; *VAS*, visual analog scale

## Discussion

### Cost-Effectiveness Challenges in Regenerative Care

With any pioneering technique, there is a natural tension between innovation, which is largely driven by the desire for improved patient outcomes, and affordability. Affordability goes beyond the healthcare system’s ability and willingness to pay and includes upstream costs such as high research and development costs and regulatory burdens. Innovation is exciting to physicians, but affordability governs the inroads these innovations can make. Balancing innovation with affordability requires scientific rigor and medical ethics, so that we can find ways to bring the safest and most effective treatments to most people.

The known advantages of regenerative approaches to spinal pain are significant and long-lasting results with few side effects or complications. Compared to pharmacologic options for spinal pain, regenerative medicine may be disease modifying and potentially curative [49]. Higher upfront costs must be balanced against a single procedure offering long-term results. Limited data on regenerative procedures to manage spinal pain and a lack of head-to-head comparative studies of regenerative versus surgical procedures makes cost analysis difficult.

Cost-effectiveness analysis (CEA) is a comparative study, in which costs of treatment options are measured against effectiveness in terms of outcomes [50]. Such analyses often weight quantitative data, such as costs, against qualitative results, such as reduced pain or disability. This heterogeneous comparison makes these studies inherently subjective, not to mention complex. Even assessing quantitative variables can be challenging, because the costs of the intervention must be defined and consistently applied across treatments and health systems. Optimally, these costs will capture both direct costs (procedural costs, imaging studies, hospitalization, drugs) as well as indirect costs (lost productivity). Less tangible costs, such as pain, suffering, and disability must be objectively quantified. Outcomes can be measured in multiple ways including pain and function scores, life years gained, quality-adjusted life years (QALYs), and reduction of disease. Overall, the cost-effectiveness ratio is the cost of the intervention divided by the benefits of the outcome(s).

A primary challenge for regenerative procedures to reduce spinal pain is the fact that these procedures are not FDA approved, so the cost data are not uniform and may not always be obtainable for research. Data availability emerges as an important shortfall in many CEA studies, particularly for regenerative spinal procedures [51]. Furthermore, it is often difficult to adequately account for things like a greater ability to pursue everyday activities, which can be very meaningful to patients but may be overlooked in the clinical setting. More and reliable data from regenerative procedures to reduce spinal pain may allow for more and more robust CEA studies, which in turn, can provide vital information to the healthcare system [52].

The role of regenerative medicine in treating spinal pain holds particular value in aging societies, such as the United States, Japan, and Western European nations, where the number of low back pain patients will likely increase. Low back pain may affect up to 75% of the geriatric population [53]. Regenerative approaches may be particularly well suited to this older population who are more likely to have comorbid conditions and polypharmacy, both of which can complicate or limit conventional surgical and pharmacologic interventions [54].

### Regenerative Medicine Within Pain Management

Interventional pain guidelines recognize regenerative medicine, including PRP and MSC, as a part of an evolving treatment landscape for pain, although recommendations remain consensus-based due to the limited high-certainty evidence [55]. Evidence from guideline-level synthesis shows that intradiscal biologic therapies are supported by evidence, with moderate clinical recommendations despite variability in the study qualities [55]. Systematic meta-analysis data also demonstrates that MSCS therapy may decrease pain scores and enhance functional status, supporting its role as an alternative to traditional symptom based pain management strategies [56].

In the context of pain management, regenerative medicine clinical improvements are relevant because treatment selection is often driven by efficacy, and also durability of effective and the cost burden associated with repeated procedures and chronic pharmacologic therapy. By highlighting the ability of regenerative medicine to be an adjunct, or alternative treatment to the traditional approaches in pain medicine, it allows the cost burden to be understood in greater context for treating the patient.

Long term observational data also suggest that intradiscal MSC therapy can produce improvements in pain and function over multiple years, which reduces the reliance on repeated interventional pain procedures [57]. Feasibility and clinical outcome data also indicates that MSC-based interventions can contribute to reduced analgesic use, including decreased dependency on medication, along with improved return-to-work outcomes [58]. Across chronic non cancer pain conditions, for example, PRP injections demonstrated statistically significant reduction in the pain score compared to a placebo, and comparative analysis also showed PRP providing greater pain relief than a traditional corticosteroid in certain pain conditions, which supports regenerative medicines role as being a longer acting pain relief in relation to conventional injection based pain therapies [59].

The durability and utilization of regenerative medicine is central to the cost effectiveness considerations in pain medicine, where interventions such as repeated injections, or chronic medication use, contribute to long term healthcare expenditures [60]. Therapies, accordingly, that reduce procedural frequency or delay progression of a disease may be meaningful in overall cost burden in healthcare, even though the initial procedural costs are higher.

Regenerative approaches are emerging as potential adjuncts or alternatives to current treatment [61]. Within this framework, regenerative medicine is a value-based intervention whose role should be evaluated in terms of clinical durability and healthcare utilization costs within the established pain care pathways.

### Evidence Gaps and Future Research Directions

The lack of head-to-head comparison studies is a shortfall in analyzing the effectiveness of regenerative approaches to the treatment of spinal pain. It is difficult to state whether a specific biologic product or certain surgical approach is preferable for a patient with a given condition. Evidence-based guidelines are lacking and there is little in literature that physicians might use to guide treatment choices. Further study is urgently warranted, because regenerative approaches hold such promise considering the increasing societal burden of low back pain.

The field of regenerative approaches for treatment of spinal pain has several open issues. While there is clinical evidence for the safety and efficacy of these procedures, this body of evidence could be expanded, with more comparative trials which would allow for better insight into the most appropriate uses of regenerative treatments. These procedures are relatively new, and more long-term data would be helpful. Finally, additional cost-effectiveness analyses and cost utility metrics would be helpful in allowing clinicians, healthcare systems, and payors to define the appropriate role for regenerative approaches to treat spinal pain as compared to the existing therapeutic modalities.

While there are no cost comparative studies of patients with specific types of back pain randomized for treatment with conventional versus regenerative approaches, the VAST trial enrolled 218 patients with chronic low back pain secondary to single-level or two-level degenerative disc disease; patients were randomized and blinded to receive intradiscal injections of viable disc allograft or saline and then crossed over to the nonsurgical management group to continue treatment. At six and 12 months, both groups achieved clinically meaningful improvements in pain and disability scores. Defining response as a reduction on the Oswetry Disability Index of ≥ 15 points, at 12 months, there were significantly more allograft patients who could be labeled responders than the saline group (76.5% vs. 56.7%, *p* = 0.03) [62].

The present investigation has several limitations. The body of literature currently necessitates a narrative rather than systematic review and there are few large, high-quality studies of the costs of regenerative medicine treatments specifically addressing spinal pain. The approaches, substances, and patient populations are heterogenous, so it is not possible to assert that results from one study can be generalized to broader groups or wider indications. Since regenerative medicine for control of spinal pain is not yet approved in many regions, cost data are largely incomplete and may be potentially misleading, as the prices may be driven availability or other factors and may not reflect costs should these procedures become approved or more widely available. The lack of uniformity across healthcare centers means that cost data will vary and could skew results. The topic selection of our research was narrow, which limited the results.

Thus, any cost-effectiveness assessments must be viewed within these limitations. Nevertheless, it is apparent that regenerative approaches to musculoskeletal disorders, including spinal pain, hold great promise in terms of potential efficacy and pain improvement. In this regard, regenerative approaches may vastly improve clinical care in coming years for patients who suffer from low back pain and a variety of other nerve and musculoskeletal disorders [63–69].

## Conclusions

Regenerative treatments for spinal pain represent a potential advance in the treatment of low back pain, a prevalent condition for which treatment can be challenging. Regenerative approaches using PRP, MSC, and other biologics can be used early in the disease process and may have disease-modifying attributes. Studies to date have been limited and small but show great promise that regenerative medicine may be an effective treatment of certain types of low back pain, offering durable results and very few side effects. Cost-effectiveness studies, particularly comparative studies, are few but demonstrate that these procedures may offer important benefits at reasonable costs. The analysis of these costs may require a longer-term evaluation allowing for high upfront costs and durable clinical benefits which result in optimal long-term benefits. Regenerative medicine has the potential to be a promising new approach to the age-old problem of lower back pain.

## Key References

- D'Souza RS, Her YF, Hussain N, Karri J, Schatman ME, Calodney AK, et al. Evidence-Based Clinical Practice Guidelines on Regenerative Medicine Treatment for Chronic Pain: A Consensus Report from a Multispecialty Working Group. J Pain Res. 2024;17:2951–3001.
  - ⚬ This supports the premise that regenerative medicine guidelines support the idea that biologics may improve pain and function, which is clinical rationale for why regenerative medicine is an emerging new field. 
- Navani A, Ambach M, Calodney A, Rosenthal R, Li G, Mahoney CB, et al. The Safety and Effectiveness of Orthobiologic Injections for Discogenic Chronic Low Back Pain: A Multicenter Prospective, Crossover, Randomized Controlled Trial with 12 Months Follow-up. Pain Physician. 2024;27(1):E65–e77.
  - ⚬ This reference addresses low back pain, which is discussed throughout the review, and it provides evidence for the safety and effectiveness of regenerative medicine, which is important to consider before thinking of cost-effectiveness.
- Beall DP, Davis T, DePalma MJ, Amirdelfan K, Yoon ES, Wilson GL, et al. Viable Disc Tissue Allograft Supplementation; One- and Two-level Treatment of Degenerated Intervertebral Discs in Patients with Chronic Discogenic Low Back Pain: One Year Results of the VAST Randomized Controlled Trial. Pain Physician. 2021;24(6):465–77.
  - ⚬ This trial provided evidence that regenerative medicine interventions outperform controls in low back pain, which provides more towards the argument of using regenerative medicine.

## Data Availability

No datasets were generated or analysed during the current study.
